# Antioxidant activities of ethanol extracts and fractions of *Crescentia cujete* leaves and stem bark and the involvement of phenolic compounds

**DOI:** 10.1186/1472-6882-14-45

**Published:** 2014-02-04

**Authors:** Nandita Das, Md Ekramul Islam, Nusrat Jahan, Mohammad Saiful Islam, Alam Khan, Md Rafikul Islam, Mst Shahnaj Parvin

**Affiliations:** 1 Department of Pharmacy, Rajshahi University, Rajshahi 6205, Bangladesh; 2 Department of Pharmacy, Int. Islamic University of Chittagong, Chittagong, Bangladesh

**Keywords:** Calabash tree, Oxidative stress, Crude extracts, Free radicals, Anti-aging

## Abstract

**Background:**

Antioxidant compounds like phenols and flavonoids scavenge free radicals and thus inhibit the oxidative mechanisms that lead to control degenerative and other diseases. The aim of this study was to investigate the antioxidant activity *in vitro*, total phenolic and flavonoid contents in ethanol extracts and fractions of *Crescentia cujete* leaves and stem bark.

**Methods:**

*Crescentia cujete* leaves and bark crude ethanol extract (CEE) and their partitionates petroleum ether (PEF), chloroform (CHF), ethyl acetate (EAF) and aqueous (AQF) were firstly prepared. Different established testing methods, such as 1, 1-diphenyl-2-picryl hydrazyl (DPPH) radical, ferric reducing power (FRP), and total antioxidant capacity (TAC) assays were used to detect the antioxidant activity. Further, the total yield, total phenolic (TPC) and total flavonoid contents (TFC) of CEE and all the fractions were determined. Ethanol extracts of both leaves and stem bark were also subjected to preliminary phytochemical screening to detect the presence of secondary metabolites, using standard phytochemical methods (Thin layer chromatography and spray reagents).

**Results:**

Phytochemical screening of crude ethanol extract of both leaves and stem bark revealed the presence of steroids, flavonoids, saponins, tannins, glycosides and terpenoids. All the fractions and CEE of leaves and bark exhibited antioxidant activities, however, EAF of leaves showing the highest antioxidant activity based on the results of DPPH, FRP and TAC assay tests. The above fraction has shown the significant DPPH scavenging activity (IC_50_ = 8.78 μg/ml) when compared with standard ascorbic acid (IC_50_ =7.68 μg/ml). The TAC and FRP activities increased with increasing crude extract/fractions content. The TPC (371.23 ± 15.77 mg GAE/g extract) and TFC (144.64 ± 5.82 mg QE/g extract) of EAF of leaves were found significantly higher as compared to other solvent fractions for both leaves and bark. TPC were highly correlated with the antioxidant activity (R^2^ = 0.9268 and 0.8515 in DPPH test for leaves and bark, respectively).

**Conclusion:**

The results of the study show that leaves of *C. cujete* possesses significant free radical scavenging properties compared with stem bark and a clear correlation exists between the antioxidant activity and phenolic content.

## Background

Plants are the source of energy for the animal kingdom and are commonly used in treating or preventing specific ailments or diseases and are considered to play a beneficial role in health care. Reactive oxygen species (ROS) such as superoxide anion (O_2_^•-^), hydroxyl radicals (OH^•^), singlet oxygen (^1^O_2_) and hydrogen peroxide (H_2_O_2_) have the damaging effects on cells [1]. Antioxidant compounds present in many plants can protect cells against this damaging caused by ROS. Cancer, cardiovascular diseases, diabetes, inflammation, degenerative diseases, anemia, ageing, and ischemia are common complicated illnesses in human directly or indirectly affected by ROS [2]. Antioxidant therapy has gained utmost importance in the treatment of these diseases and oxidative damage. However, the factors such as high cost, lack of availability and side effects of synthetic antioxidants remain as major setbacks in combating oxidative stress. For example, BHT has been suspected to be carcinogenic.

In this direction, natural antioxidants received a prominence as they are often free from side effects, less expensive and abundant in many plant sources [2]. Large number of medicinal plants has been investigated for their antioxidant properties. Natural antioxidants either in the form of raw extracts or their chemical constituents are very effective to prevent the destructive processes caused by oxidative stress [3,4]. More recently, it has become evident that phenolic natural products may reduce oxidative stress by indirect antioxidant action [5-7]. Human body has an inherent antioxidative mechanism and many of the biological functions such as the antimutagenic, anticarcinogenic, and antiaging responses originate from this property [8,9]. Antioxidants stabilize or deactivate free radicals, often before they attack targets in biological cells [10]. Over 50% of the drugs in clinical trials for anticancer activity were isolated from natural sources or related to them [11] also been reported with antioxidant property. Luteolin, kaempferol, quercitrin, rutin, myricetin, and vitamin C are powerful antioxidants that inhibit the oxidation of low-density lipoprotein (LDL), a major factor in the promotion of atherosclerosis that can lead to heart attack or stroke. Phenolic acids, flavonoids, stilbenes and lignans are the most abundantly occurring polyphenols in plants that reduce the risk of cancer, act against allergies, ulcers, tumors, platelet aggregation and are also effective in controlling hypertension [12-14]. Limonoids, the second major subclass of terpenoids, are the biologically active phytochemicals present in citrus which act as antioxidant and protect lung tissues from free oxygen radicals. *In vitro* studies show that antioxidant nomilin and limonoid glycosides have significant ability to inhibit proliferation of human breast cancer [15,16]. Hence, investigations in naturally occurring antioxidants has considerably increased and natural products have regained prominence in the recent past with increasing understanding of their biological significance such as antioxidant, radical scavenging activities and increasing recognition of the origin and function of their structural diversity [17-20]. It then becomes necessary to search new source for noble antioxidants, especially those that would be safe and cheap and thus easily affordable by all population. The present study was designed to investigate the total phenolic contents (TPC) and total flavonoid contents (TFC) to evaluate the antioxidant activities of the various fractions of ethanol extract of leaves and stem bark of *Crescentia cujete.*

Calabash tree, scientifically known as *Crescentia cujete* belongs to the family of Binoniaceae and is also known as the gourd tree. The tree is 6 to 10 m tall with a wide crown and long branches covered with clusters of tripinnate leaves and gourd-like fruit. The branches have simple elliptical leaves clustered at the anode. According to folk medicine, the fruit pulp is used for respiratory problems such as asthma and also used as laxative. The bark is used for mucoid diarrhea. Bark decoction used to clean wounds and pounded leaves used as poultice for headaches. Internally, leaves used as diuretic and also used to treat hematomas and tumors. Fruit decoction used to treat diarrhea, stomachaches, cold, bronchitis, cough, asthma, and urethritis. The leaves are also used for hypertension [21]. The juice from fruits mixed with sugar and/or bee's honey and eaten for problems of the respiratory system (asthma, catarrh), the digestive system (stomach pains, intestinal parasites) and the female reproductive apparatus (infertility) [22]. Naphtoquinones [23], iridoid glycosides, aucubin, plumieride, and asperuloside [24] have been reported as the constituents of the leaves of this plant. Plant principally contains tartaric acid, cianhidric, citric acid, crescentic acid, tannins, beta-sitosterol, estigmasterol, alpha and beta amirina, estearic acid, triacontanol, palmitic acid, flavonoids-quercetin, apigenin, 3-hydroxyoctanol glycosides and p-hydroxybenzoyloxy glucose [25,26]. Hexane, ethyl acetate, methanol and water extracts of *C. cujete* leaves were investigated against bacterial strains [27]. DPPH radical scavenging, antioxidant activity by β-carotene bleaching test and cytotoxic activity of the methanol extract of aerial parts of this plant were evaluated by David *et al.*[28]. However, according to our knowledge, detailed antioxidant activities on different fractions of leaves and stem bark extracts have not been studied, yet. In this study, we report the TPC and TFC as well as the antioxidant properties of *C. cujete* in different plant parts (leaves and stem bark) to identify the plant part and solvent extract that gives the highest antioxidant activities and this may justify important ethnomedical uses of this medicinal plant as antioxidants have diverse biological actions.

## Methods

### Plant material

*C. cujete* leaves and bark samples were collected from University of Rajshahi, Rajshahi, Bangladesh. Identification of the plant was confirmed by Department of Botany, University of Rajshahi and a voucher specimen has been deposited in the departmental herbarium with accession no. PH-115. The collected plant parts were dried for one week and pulverized into a coarse powder using a suitable grinder. The powder was stored in an airtight container and kept in a cool, dark, and dry place.

### Extract preparation

Approximately 450 g of powdered leaves and 380 g of powdered bark was placed separately in a clean, flat-bottomed glass container and soaked in ethanol. The container with its contents was sealed and kept for 7 days accompanied by occasional shaking and stirring. The entire mixture then underwent a coarse filtration by a piece of clean, white cotton material. The extract then was filtered through Whatman filter paper (Bibby RE200, Sterilin Ltd., UK). After filtration, the filtrate was evaporated to dryness at 50°C under reduced pressure using a rotary evaporator to obtain the ethanol crude extract (9.5 g for leaves and 8.5 g for bark). The crude ethanol extract (CEE) was suspended with distilled water (150 ml) and partitioned with petroleum ether, chloroform and ethyl acetate. The resultant partitionates i.e., petroleum ether (PEF), chloroform (CHF), ethyl acetate (EAF) and water (AQF) soluble fractions (Table 1) were used for the biological screenings except PEF.

**Table 1:** **Extraction yield of various fractions of *C. cujete *** **leaves and bark**

**Leaves**	**Extraction yield (%)**	**Bark**	**Extraction yield (%)**
PEF	25%	PEF	28.23%
CHF	12.5%	CHF	12.94%
EAF	12.5%	EAF	14.12%
AQF	37.5%	AQF	34.12%

PEF, petroleum ether fraction; CHF, chloroform fraction; EAF, ethyl acetate fraction; AQF, water fraction.

### Chemicals

1,1-diphenyl-2-picrylhydrazyl (DPPH) and gallic acid were purchased from Sigma-Aldrich USA. Folin-Ciocalteu was obtained from Merck (Damstadt, Germany). potassium ferricyanide, potassium acetate, phosphate buffer, ferrous ammonium sulphate, ascorbic acid (AA), aluminium chloride (AlCl_3_), trichloro acetic acid (TCA), sodium phosphate, ammonium molybdate, tannic acid, quercetin, acetyl acetone and ferric chloride (FeCl_3_) were purchased from Sigma Chemical Co. (St. Louis, MO, USA). All the chemicals used in the study were of analytical grade.

### Phytochemical screening of ethanolic extracts

Small amount of crude ethanol extract of *C. cujete* leaves (also done for stem bark) was dissolved in a suitable solvent and applied as small spot on the activated thin layer chromatography (TLC) plate. The resulting plate runs with solvent systems (100% chloroform, 70% chloroform + 30% n-hexane, 50% chloroform + 50% n-hexane, 30% chloroform + 30% n-hexane + 40% methanol) and visualized with various spray reagents (vanillin-sulfuric acid spray, ceric sulfate-sulfuric acid spray, Dragendorff’s spray, aluminium chloride spray, 4-aminoantipyrine/potassium hexacyanferrate (III) spray, p-anisaldehyde–sulfuric acid spray, ethanolamine diphenylborate, chloranil reagent spray) to determine the presence of various classes of active chemical constituents such as alkaloids, glycosides, steroids, flavonoids, saponins, tannins and terpenes using standard procedures [29,30].

### Antioxidant assay

#### ***DPPH free radical scavenging assay***

The free radical scavenging activity of the extracts as well as their various fractions was evaluated according to described methods [31,32]. Briefly, sample solution with different concentrations (ranging from 0 to 200 μg/ml) was mixed with 0.3% of DPPH methanol solution. The reaction mixtures were incubated at room temperature and allowed to react for 30 minutes in the dark. After 30 min, the absorbance values were measured at 517 nm and converted into percentage of antioxidant activity. Ascorbic acid (AA) was used as a positive standard control. The percentage of inhibition of DPPH (%) was calculated as follows:

$$\text{\% inhibition of DPPH}=\frac{\text{Absorbance of control - Absorbance of test sample}}{\text{Absorbance of control}}\times 100$$

The concentration of sample required to scavenge 50% of the DPPH free radical (IC_50_) was determined from the curve of % inhibitions plotted against the respective concentration.

#### ***Ferric reducing power assay***

The reducing power of the leaves and bark was determined according to method as previously described [33]. Aliquot (0.25 ml) of samples solution at different concentrations (ranging from 12.5 to 100 μg/ml) was mixed with 0.625 ml of 0.2 M phosphate buffer (pH 6.6) and 0.625 ml of 1% (w/v) solution of potassium ferricyanide. After mixing well, all the mixtures were incubated in a water bath at 50°C for 20 min. Then, 0.625 ml of 10% (w/v) TCA solution was added and the mixture was then centrifuged at 3000 rpm for 10 min. A 1.8 ml of the supernatant was combined with 1.8 ml of distilled water and 0.36 ml of a 0.1% (w/v) solution of ferric chloride. The absorbance was measured at 700 nm with a spectrophotometer. Ascorbic acid was used as positive control. All the tests were run in triplicate and results were reported as mean ± SD.

#### ***Phosphomolybdate radical scavenging activity***

The assay was based on the reduction of Mo(VI)-Mo(V) by the extracts and subsequent formation of a green phosphate/Mo(V) complex at acidic pH [34]. Each sample (0.1 ml) was mixed with 3 ml of reagent solution (0.6 M sulphuric acid, 28 mM sodium phosphate and 4 mM ammonium molybdate). The tubes were incubated at 95°C for 90 min. The mixture was cooled to room temperature and the absorbance of the solution was measured at 695 nm against a blank. The assays were carried out in triplicate and expressed as mean ± SD. The antioxidant activity was expressed as the absorbance of the sample.

#### ***Determination of total phenolic content***

The concentrations of phenolic compounds in the samples of *C. cujete* leaves and bark were measured according to the Folin-Ciocalteu method [35]. Briefly, the samples solution (0.5 ml) at different concentrations (ranging from 100 to 1100 μg/ml) was mixed with 2.58 ml of Folin-Ciocalteu’s phenol reagent. After 3 min, 0.3 ml of saturated sodium carbonate solution was added to the mixture. The reaction mixtures were incubated at room temperature (25°C) for 20 min. The absorbance was measured at 760 nm with a spectrophotometer. Gallic acid solutions with concentrations ranging from 25 to 400 μg/ml were used for calibration. A dose response linear regression was generated by using the gallic acid standard absorbance and the levels in the samples were expressed as gallic acid equivalents (mg of GAE/g of extract). The estimation was performed in triplicate, and the results were expressed as mean ± SD.

#### ***Determination of total flavonoid content***

The total flavonoid content was estimated by aluminium chloride method [36]. Plant samples (0.5 ml) were mixed with 2.5 ml of distilled water and 150 μl NaNO_2_ solution (5%). The contents were vortexed for 10 sec and left at room temperature for 5 min. Then, 300 μl AlCl_3_ (10%), 1 ml NaOH (1 mM) and 550 μl of distilled water were added. The solution was mixed well and kept for 15 min. The absorbance for each sample was measured at 510 nm. Quercetin concentrations ranging from 25 to 400 μg/ml were prepared and the standard calibration curve was obtained. The total flavonoid content was calculated using standard quercetin calibration curve. The results were expressed as milligrams of quercetin equivalents (QE) per gram of extract.

#### ***Statistical analysis***

The statistical analyses were performed by a one-way ANOVA and the Student’s t-test. Free R-software version 2.15.1 (http://www.r-project.org/) and Microsoft Excel 2007 (Roselle, IL, USA) were used for the statistical and graphical evaluations. The results were expressed as mean ± SD from three separate observations.

## Results

### Phytochemical screening

The phytochemical screening of CEE of leaves and stem bark showed the presence of different types of secondary metabolites, namely saponins, alkaloids, tannins, glycosides, terpenes and flavonoids. These phytocompounds were present in both the extracts. However, our tests were showing the absence of alkaloids in CEE of leaves.

### DPPH free radical scavenging assay

**Figure 1:**
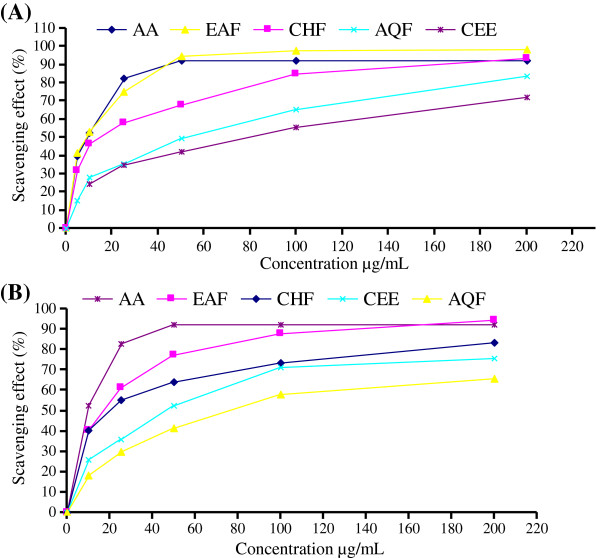
**Scavenging activity (%) on DPPH radicals of (A) CEE and its fractions of leaves (B) CEE and its fractions of bark.** Each value is expressed as mean ± SD (n = 3).

The DPPH radical scavenging activity of leaves and stem bark were shown in Figure 1 and Table 2 as comparable with known antioxidant AA. Figure 1(A) showed the scavenging effects of CEE of leaves and its various fraction on DPPH radical and were in the following order: EAF > CHF > AQF > CEE. At a concentration of 100 μg/ml, the values for % scavenging of DPPH for leaves extract and fractions ranged between 55.32-97.73% and values for IC_50_ was in the range 8.78 to 80.21 μg/ml. The scavenging effects of crude ethanol extract of bark and its fractions, as shown in Figure 1(B), were in the order of EAF > CHF > AQF > CEE. Comparatively lower activity was observed with stem bark crude ethanol extract and fractions where the % scavenging of DPPH ranged between 58.19 to 87.59% and IC_50_ from 18.34 to 74.69 μg/ml, while at the same concentration that of AA was 92.12% with IC_50_ of 7.68 μg/ml (Table 2).

**Table 2:** **IC**_**50 **_**values (μg/mL) of crude ethanol extract and derived fractions of *C. cujete *** **leaves and bark in DPPH free radical scavenging evaluation assay**

**Extract/Fractions/Chemical**	**Leaves**	**Bark**
CEE	80.21	45.78
CHF	15.56	20.12
EAF	8.78	18.34
AQF	52.43	74.69
Standard (AA)	7.68	7.68

CEE, crude ethanol extract; CHF, chloroform fraction; EAF, ethyl acetate fraction; AQF, water fraction.

### Reducing power activity

**Figure 2:**
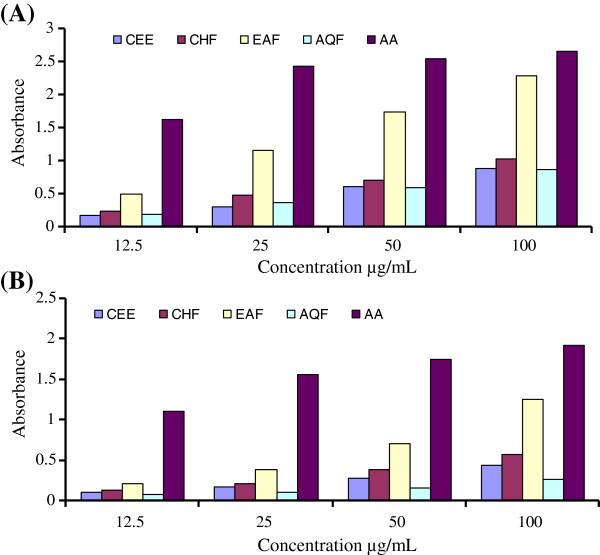
**Ferric reducing power assay. (A)** CEE of leaves and its fractions, CHF, EAF and AQF **(B)** CEE of bark and its fractions, CHF, EAF and AQF. Reducing power was measured at different concentration of extracts (12.5–100 μg/ml). AA was used as control. The results are expressed as mean ± SD of three replicates.

Figure 2 showed the reducing power of leaves and stem bark as a function of concentration of antioxidant compounds. The reducing power of the extracts and their fractions increased gradually with the increase in concentrations. Comparatively better activity was observed in leaves. Amongst the extract/fractions of leaves, the highest activity was found in EAF followed by CHF, AQF and CEE as shown in Figure 2(A). At highest test concentration (100 μg/ml) the absorbance of EAF was 2.296 as compared with the absorbance of AA (2.654) while rest of the fractions and CEE showed absorbance in the range of 0.878-1.032. Reducing power of bark extract/fractions at all test concentrations was lower than the activities of leaves extract/fractions. At highest test concentration (100 μg/ml) the absorbance of CEE and fractions of stem bark were found in the range 0.27-1.255 (Figure 2B).

### Phosphomolybdate radical scavenging activity

**Figure 3:**
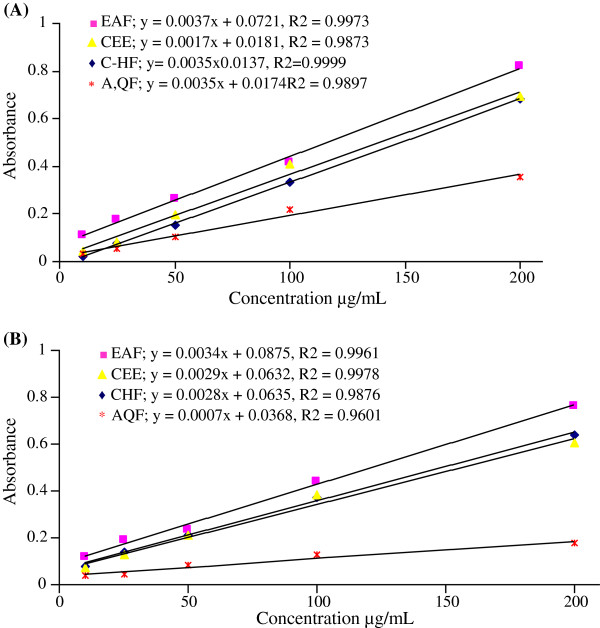
Correlation between different concentrations of extract/fractions and their total antioxidant capacity as determined by the formation of phosphomolybdenum complex assay (A) Leaves (B) Bark.

The total antioxidant capacity was measured by phosphomolybdenum method. Organized in decreasing order of TAC, the CEE and fractions of leaves ranked as follows: EAF > CHF > CEE > AQF, whereas that of bark was EAF > CHF > CEE > AQF. The crude extracts and fractions of leaves and bark were found to increase the TAC with the increasing concentration of the samples (Figure 3). The correlation coefficient (R^2^) between extracts/fractions and the formation of phosphomolybdenum complex were also shown in Figure 3(A) and Figure 3(B).

### Determination of total phenol and flavonoid content

The TPC and TFC of CEE and various fractions of leaves and bark were shown in Table 3. All the samples contained a considerable amount of phenol and flavonoids. TPC of crude ethanol extract and fractions of *C. cujete* leaves varied widely, ranging from 28.07 ± 9.47 mg to 371.23 ± 15.77 mg GAE/g of extract, whereas that of bark ranging from 61.18 ± 5.706 mg to 326.75 ± 4.659 mg GAE/g of extract. Flavonoid contents also varied widely among different fractions of bark. The highest amount was found in EAF (82.7 ± 9.03 mg of GAE/g of extract), followed by CHF (42.62 ± 6.46 mg of GAEs/g of extract), and CEE (20.77 ± 3.41 mg of GAE/g of extract) extracts in the decreasing order. AQF did not show any response at that concentration. Highest phenolics and flavonoids content were found in EAF of leaves.

**Table 3:** **Total phenolic and flavonoid content in CEE and fractions of leaves and bark of *C. cujete***

**Extract/Fractions**	**Leaves**		**Bark**	
	**TPC**	**TFC**	**TPC**	**TFC**
CEE	28.07 ± 9.47	139.57 ± 3.75	111.43 ± 2.406	20.77 ± 3.41
CHF	247.56 ± 7.58	101.20 ± 5.37	234.83 ± 6.608	42.62 ± 6.46
EAF	371.23 ± 15.77	144.64 ± 5.82	326.75 ± 4.659	82.7 ± 9.03
AQF	114.46 ± 29.41	16.04 ± 3.23	61.18 ± 5.706	-

Phenolics are represented as mg of GAE/g of extract sample. Values are expressed as mean ± SD of three replicates. CEE, crude ethanol extract; CHF, chloroform fraction; EAF, ethyl acetate fraction; AQF, water fraction.

### The correlation between total phenols content and antioxidant activity (DPPH)

**Figure 4:**
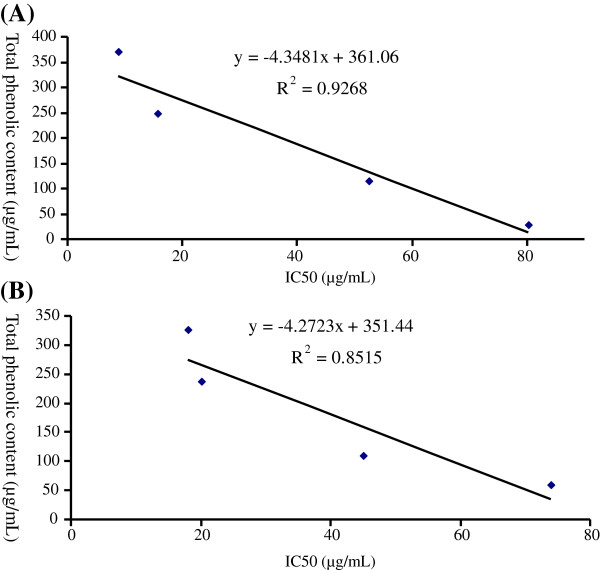
**Correlation of total phenolic contents with IC**_**50 **_**of DPPH free radical scavenging (A) leaves (B) Bark.**

Figure 4 showed the correlation of total phenolic content with DPPH scavenging assay. The relationship between free radical scavenging activity (Y) with total phenolic contents (X) for leaves and bark revealed coefficient of determination (R^2^) of 0.9268 and 0.8515, respectively.

## Discussions

To the best of our knowledge present study was the first attempt to evaluate the ability of the leaves and stem bark CEE and various fractions to act as antioxidant agents. The most natural antioxidants are multifunctional. Therefore, a reliable antioxidant evaluation protocol requires different antioxidant activity assessments to account various mechanisms of antioxidant action. In this study, several techniques have been used to determine the *in vitro* antioxidant activity to allow rapid screening of substances.

### DPPH radical scavenging activity

DPPH radical scavenging model is widely used method to evaluate antioxidant activity of natural compound and plant extracts. The degree of discoloration indicates the scavenging potential of the antioxidant extract, which is due to the hydrogen donating ability [37]. The experimental data revealed that all the fractions and crude ethanol extracts of leaves and stem bark have the effects of scavenging free radicals and a dose dependent relationship in the DPPH radical scavenging activity. IC_50_ value of 10.3 mg/ml for DPPH radical scavenging has been reported for the methanol extract of the aerial part of this plant [28]. In this study, EAF of leaves exhibited strong DPPH radical quenching activity (IC_50_ = 8.78 μg/ml), higher than the reported assay and is also very close to the standard AA. Presence of flavonoids-quercetin, apigenin, naphthoquiones, tannins and steroids in *C. cujete* might contribute towards the DPPH radical scavenging activity since these classes of compounds are known as free radical scavenger [38,39]. The involvement of free radicals, especially their increased production leads to the development of cardiovascular diseases and cancer. Thus, the consumption of *C. cujete* leaves can be beneficial in preventing oxidative stress related numerous chronic diseases.

### The reducing power capacity

The reducing power of the CEE and all fractions was determined by direct electron donation in the reduction of ferri cyanide [Fe(CN)6]^3-^ to ferro cyanide [Fe(CN)6]^4-^. The product was visualized by addition of free Fe3^+^ ions after the reduction reaction, by forming the intense Prussian blue colour complex, (Fe3^+^)_4_[Fe^2+(^CN^-^)_6_]^3^, and quantified by absorbance measurement at 700 nm [40]. The presence of reductants (i.e. antioxidants) in *C. cujete* leaves and stem bark cause the reduction of the Fe3+ /ferricyanide complex to the ferrous form which was monitored by measuring the formation of Perl’s Prussian blue at 700 nm. Figure 2 shows the reductive capabilities of various parts of the *C. cujete* extracts compared to ascorbic acid. Generally, the EAF of *C. cujete* leaves showed a significantly higher reducing power at all the tested concentrations compared to the other extract and fractions of stem bark. Previous studies have pointed out that the phenolic compounds play an important role in the reducing power of the extracts [41]. Therefore, like the DPPH radical scavenging activity, the observed reducing power of leaves and stem bark was in agreement with the chemical constituents in the extracts/fractions of *C. cujete*.

### The total antioxidant capacity

The total antioxidant capacity is based on the reduction of molybdenum (VI) to molybdenum (V) by extracts and subsequent formation of a green phosphate/molybdenum (V) complex at acidic pH. The high absorbance values indicated that the sample possessed significant antioxidant activity. The EAF of leaves had significant total antioxidant activity and the effect increased with increasing concentration (Figure 3). A strong correlation coefficient (R^2^) between extracts/fractions of leaves and stem bark and the formation of phosphomolybdenum complex was observed (Figure 3). The difference in the amount of antioxidant of extracts/fractions may be attributed to the differences in the amount and kind of existing antioxidant compounds in them such as carotenoids, phenol and ascorbic acid [42]. The antioxidant activity shown by the leaves and stem bark may be due to the presence of tannins, terpenoids, steroids and flavoniods.

### Polyphenols (TPC and TFC) contents

Polyphenols were found in all the extracts/fractions of leaves and stem bark. The obtained results for DPPH are in agreement with the phenol contents determined for each sample. Plant polyphenols are produced from phenylalanine or from its precursor shikmic acid. These phenolics are important dietary antioxidants because they have ideal structural chemistry for free radical scavenging activities, and have been shown to be more effective antioxidants *in vitro* than vitamins E and C on a molar basis [43]. Polyphenols exhibit a wide range of biological effects such as protection of LDL oxidation *in vivo* with significant consequences in atherosclerosis and also protect DNA from oxidative damage with important consequences in the age-related development of some cancers [44]. Our findings suggested that leaves and stem bark of *C. cujete* rich in phenolic and flavonoid contents which are the major contributor to scavenge the free radicals in oxidation pathways.

The results obtained from correlation between polyphenols (phenol and flavonoid) and DPPH scavenging suggested that phenolic compounds are dominant contributors to the antioxidant activity of the extract/fractions. It is also reported that secondary metabolites in the extracts such as polyphenols, phenolic acids, flavonoids, flavonols, diterpenes, tannins, phytosterols, fatty acid esters, phenylpropanoids, alkaloids and glycosides are important classes of bioactive compounds which have great importance in medicinal chemistry and natural product research for their high antioxidant properties [39].Our preliminary phytochemical screening of leaves and stem bark also revealed the presence of above class of compounds. Therefore, the antioxidant activity may also come from other antioxidant present in the fractionated extract as well.

## Conclusion

The replacement of synthetic with natural antioxidants (because of implications for human health) is advantageous. In the present study, analysis of free radical scavenging activity and total phenolic and flavonoid contents showed that mainly the EAF of *C. cujete* leaves can be the potent source of natural antioxidants. However, further detailed investigation, especially *in vivo* antioxidant and toxicity studies are needed to justify its use as a natural source of antioxidants to prevent the progression of many diseases.

## Abbreviations

CEE: Crude ethanol extract; PEF: Petroleum ether fraction; CHF: Chloroform fraction; EAF: Ethyl acetate fraction; AQF: Water fraction; AA: Ascorbic acids; GAE: Gallic acid equivalent; QE: Quercetin equivalent; SD: Standard deviation.

## Competing interests

The authors declare that they have no competing interests.

## Authors’ contributions

ND, prepared the extracts and carried out all the experimental process. MSP, designed the current project, supervised the work and wrote the manuscript. MEI, worked closely with ND in the laboratory to carry out the experiments and helped in preparing the manuscript. MNJ, helped to carry out the assay. AK, MRI and SI evaluated the data and edited the manuscript. All the authors read and approved the final manuscript.

## Pre-publication history

The pre-publication history for this paper can be accessed here:

http://www.biomedcentral.com/1472-6882/14/45/prepub
